# Construction of a High-Density Genetic Linkage Map for the Mapping of QTL Associated with Growth-Related Traits in Sea Cucumber (*Apostichopus japonicus*)

**DOI:** 10.3390/biology11010050

**Published:** 2021-12-30

**Authors:** Wei Cui, Da Huo, Shilin Liu, Lili Xing, Fang Su, Hongsheng Yang, Lina Sun

**Affiliations:** 1 CAS Key Laboratory of Marine Ecology and Environmental Sciences, Institute of Oceanology, Chinese Academy of Sciences, Qingdao 266071, China; cuiwei19@mails.ucas.ac.cn (W.C.); huoda@qdio.ac.cn (D.H.); shlliu@qdio.ac.cn (S.L.); lilixing@qdio.ac.cn (L.X.); sufang19@mails.ucas.ac.cn (F.S.); hshyang@qdio.ac.cn (H.Y.); 2 Laboratory for Marine Ecology and Environmental Science, Qingdao National Laboratory for Marine Science and Technology, Qingdao 266237, China; 3 Center for Ocean Mega-Science, Chinese Academy of Sciences, Qingdao 266071, China; 4 CAS Engineering Laboratory for Marine Ranching, Institute of Oceanology, Chinese Academy of Sciences, Qingdao 266071, China; 5 University of Chinese Academy of Sciences, Beijing 100049, China; 6 Shandong Province Key Laboratory of Experimental Marine Biology, Qingdao 266071, China; 7 The Innovation of Seed Design, Chinese Academy of Sciences, Wuhan 430071, China

**Keywords:** GBS, growth traits, genetic linkage map, SNP, QTL, marker-assisted selection breeding, sea cucumber (*Apostichopus japonicus*)

## Abstract

**Simple Summary:**

Slow growth and germplasm degradation have restricted the sustainable commercial development of the sea cucumber industry. To analyze the genetic mechanism of growth traits of sea cucumbers, we constructed a high-density genetic linkage map based on single nucleotide polymorphism (SNP) molecular markers and performed a quantitative trait loci (QTL) mapping analysis. We annotated a critical candidate gene related to growth traits and explored mRNA expression levels. The results showed that the gene was significantly highly expressed during the larval developmental stages. These results can be used to genetically improve the growth traits of sea cucumbers.

**Abstract:**

Genetic linkage maps have become an indispensable tool for genetics and genomics research. Sea cucumber (*Apostichopus japonicus*), which is an economically important mariculture species in Asia, is an edible echinoderm with medicinal properties. In this study, the first SNP-based high-density genetic linkage map was constructed by sequencing 132 *A. japonicus* individuals (2 parents and 130 offspring) according to a genotyping-by-sequencing (GBS) method. The consensus map was 3181.54 cM long, with an average genetic distance of 0.52 cM. A total of 6144 SNPs were assigned to 22 linkage groups (LGs). A Pearson analysis and QTL mapping revealed the correlations among body weight, body length, and papillae number. An important growth-related candidate gene, protein still life, isoforms C/SIF type 2 (*sif*), was identified in LG18. The gene was significantly highly expressed during the larval developmental stages. Its encoded protein reportedly functions as a guanine nucleotide exchange factor. These results would facilitate the genetic analysis of growth traits and provide valuable genomic resources for the selection and breeding of new varieties of sea cucumbers with excellent production traits.

## 1. Introduction

Sea cucumber (*Apostichopus japonicus*) is an important edible aquaculture species in East Asia because of its considerable medicinal value. In 2020, more than 171,000 tons of sea cucumber were produced from a farming area of 246,745 hectares. The cultivation of sea cucumber has become one of the major mariculture industries in China. However, increases in the scale of sea cucumber farming have highlighted specific problems, including germplasm degradation, slow growth, and frequent diseases, of which slow growth is one of the most prominent problems. Therefore, enhancing sea cucumber germplasm and cultivating new varieties with improved traits (e.g., rapid growth) are critical for sustainable commercial production. The substantial limitations of traditional breeding methods have led to a greater focus on improving traits via molecular marker-assisted selection (MAS) breeding. Significant progress has been made in the development of sea cucumber genetic and genomic resources, which has laid a solid foundation for the promotion of MAS. For example, previous studies on sea cucumber have resulted in information regarding its diploid genome (2n = 44) [1]; two low-density genetic maps based on amplified fragment length polymorphisms (AFLPs), microsatellites, and single nucleotide polymorphisms (SNPs) [2,3]; a high-density map constructed according to a 2b-restriction site-associated DNA (2b-RAD) method [4]; and the release of a sequenced genome [5]. The transition from traditional breeding methods to MAS-based breeding has become an irreversible trend. However, high-density and high-resolution genetic linkage maps are prerequisites for such breeding.

A genetic linkage map constructed on the basis of gene linkages and genetic recombination rates can be used to infer the relative positions of genes or genetic markers on chromosomes, making it important for inheritance and genome research [6]. The resolution and density of the map mainly depend on the size of the populations, the number of genetic markers, and the accuracy of genotyping [4,7]. According to the development of genetic markers, the research of genetic maps can be divided into two stages: a classical genetic map, which mainly adopts morphological markers, cytological markers and biochemical markers to indirectly reflect the sequence of functional genes on chromosomes, and a molecular genetic map, which relies on DNA molecular markers to construct a map. In the mid-19th century, Mendel pioneered the application of morphological traits as genetic markers in peas. In 1919, Sturtevant [8] first constructed a linkage map of five X chromosome loci in Drosophila. In the 1980s, the application of another more critical genetic marker, the molecular marker, brought revolutionary changes to the construction of the genetic map. In the late 1990s, the first-generation genetic linkage maps for aquaculture species, including Nile tilapia (*Oreochromis niloticus*) [9] and Pacific oyster (*Crassostrea gigas*) [10], were constructed mainly on the basis of microsatellite, random amplified polymorphic DNA (RAPD), and AFLP markers. In 2003, Nichol et al. [11] used 1359 markers to construct a high-density genetic map for rainbow trout (*Oncorhynchus mykiss*) (i.e., second-generation genetic linkage map). These maps contained few markers, which were separated by relatively large distances. Traditional genotyping technology can no longer meet the requirements for constructing highly accurate high-density genetic linkage maps. High-throughput, accurate, and inexpensive genotyping methods are needed for comprehensively characterizing genetic variation. The development of next-generation sequencing technology substantially affected MAS-based research on aquaculture species.

Genotyping strategies developed on the basis of next-generation sequencing technology have been applied to minimize genomic complexity, thereby simplifying SNP detection. The commonly used methods for reduced-representation genome sequencing (RRGS) mainly include restriction site-associated DNA (RAD) and GBS. Their essence is to digest the genome and sequence the enzyme sections. The differences lie in the details of single or double enzyme digestion, whether to use ultrasonic random interruption, different restriction enzyme, adapters and so on. RAD-seq needs to be randomly interrupted by ultrasound to select the sequence, while GBS directly amplified the corresponding fragment by PCR. Genotyping-by-sequencing [12] has been used to develop molecular markers, construct high-density genetic linkage maps, analyze population genetics, and conduct genome-wide association studies. Briefly, GBS involves the digestion of the genome using appropriate restriction enzymes prior to a high-throughput, paired-end sequencing step. Choosing appropriate methylation-sensitive restriction enzymes, which can avoid repetitive genomic regions and target low-copy regions 2- to 3-times more efficiently, is the key to a successful GBS analysis. Compared with the RAD method, the GBS method is less complicated. The single-well digestion of genomic DNA and ligation of adapters result in decreased sample processing, fewer DNA purification steps, and the elimination of a fragment-size selection step [13]. In the present study, we used an F_1_ population as the experimental material to construct the first high-density *A. japonicus* genetic linkage map with 6144 SNPs by applying GBS technology. Body weight, body length, and papillae number were determined for each individual and used for quantitative trait locus (QTL) mapping. Eleven growth-related candidate genes were identified. This map is a useful resource for future research on mining of information on functional genes associated with economically important traits, as well as for implementing molecular MAS breeding of *A. japonicus.*

## 2. Materials and Methods

### 2.1. Mapping Population and DNA Extraction

A mapping population was established by mating a pair of unrelated and highly polymorphic male (MP) and female (FP) sea cucumber specimens (with a wet weight of 96 g and 235 g, body length of 17.2 cm and 24.3 cm, respectively), which were collected from a wild population in Rushan, Shandong province, China. After the fertilization, the body wall and muscle tissues of the parents were sampled and preserved in ethanol. A total of 130 randomly selected 1-year-old offspring and the parents were used for genetic mapping and QTL detection. Growth traits, including wet body weight, body length, and papillae number, were recorded for all individuals. The body wall and muscle tissues were collected from all specimens and stored at −80 °C. Genomic DNA was extracted from the frozen samples according to a traditional phenol–chloroform DNA extraction protocol [14]. The quality of the genomic DNA samples was evaluated via 1% agarose gel electrophoresis and the concentrations were determined using the NanoDrop 2000 spectrophotometer (Thermo Fisher Scientific, Wilmington, DE, USA). High-quality genomic DNA (i.e., in terms of purity and integrity; OD_260_/OD_280_ = 1.8–2.0 and OD_260_/OD_230_ = 1.8–2.0) with a concentration greater than 50 ng/μL was used to construct the GBS libraries.

### 2.2. GBS Library Construction and Sequencing

To ensure that we selected an appropriate restriction enzyme, which is critical for a successful GBS run, we conducted a digital enzyme cut analysis first. The GBS libraries were constructed for the 2 parents and 130 offspring according to a previously described standard protocol [13]. Briefly, we digested 0.1–1 μg genomic DNA using *MseI* (cut-site: TTAA), *HaeIII* (cut-site: GGCC), and *MspI* (cut-site: GGCC), which are insensitive to Dam, Dcm, and CpG methylation. Common and barcoded adapters with sticky ends were ligated to the digested fragments before the PCR amplification and Illumina sequencing. The primer sequences were as follow: Primer 1: 5′-AATGATACGGCGACCACCGAGATCTACACTCTTTCCCTACACGACGCTCTTCCGATCT-3′ and Primer 2: 5′-CAAGCAGAAGACGGCATACGAGATCGGTCTCGGCATTCCTGCTGAACCGCTCTT CCG ATCT-3′ [15]. The Illumina HiSeq 2500 sequencing platform was used for the paired-end sequencing of the GBS libraries. [5]. The raw read data were archived at the NCBI Sequence Read Archive (SRA) under Accession Number PRJNA777474. The raw reads for each sample were filtered during a quality control step to eliminate reads containing adapters and/or ambiguous bases (N) as well as low-quality reads (>50% bases harboring Phred quality score ≤ 20) [4,16,17].

### 2.3. SNP Calling and Genotyping

The retained clean reads for each individual were aligned to the *A. japonicus* genome [5] using BWA (version 0.1.22) [17]. The reads that were aligned to only one position of the reference genome were selected for subsequent analyses. The SAMtools (version 1.0) program was used for detecting SNPs and for filtering the BAM files [18]. To decrease the number of false positives, the SNP base support number for the parents and offspring was required to be no less than 4 and 1, respectively. To minimize the number of false positives caused by repeated regions, the SNP base support number was required to be no more than 1000. Additionally, the genotype quality threshold was set at ≥10. The genotype detection results for the FP and MP were used to identify the SNPs between the parents. The sites lacking parental information were filtered out, and those that were homozygous and polymorphic between the parents were retained. These filtered SNPs were categorized as follows: ‘hk × hk’ (markers in both parents), ‘lm × ll’ (markers in the MP), or ‘nn × np’ (markers in the FP). The three types of markers were further screened by checking for abnormal bases and by filtering for genotyping completeness.

### 2.4. Linkage Map Construction

Sex-specific linkage maps were constructed using the cross-pollination algorithm of JoinMap (version 4.0) [19]. A logarithm of odds (LOD) threshold of 3.0 was applied to assign markers to linkage groups (LGs). Recombination rates were calculated according to the regression mapping algorithm and converted into map distances (i.e., centimorgans (cM)) using the Kosambi mapping function. A consensus genetic linkage map was constructed using MergeMap [20] by integrating the sex-specific maps on the basis of shared markers. All genetic linkage maps were drawn using Perl SVG.

### 2.5. QTL Mapping and Potential Candidate Gene Identification

The phenotypic data (body weight, body length, and papillae number) of the 130 offspring were preprocessed using R (version 4.0.2). The growth-related QTL were mapped using the ‘interval mapping’ and ‘restricted multiple QTL model mapping’ algorithms of MapQTL (version 6.0) [21]. The LOD significance thresholds for each trait were calculated according to a permutation test (1000 permutations) at a significance level of *p* < 0.05. The QTL confidence interval was calculated as previously described [22]. The phenotypic variance explained (PVE) for each QTL was calculated on the basis of the offspring population variance. Furthermore, the QTL results and *A. japonicus* genome sequence [5] were used to identify SNP loci related to growth and for screening candidate growth-related genes.

### 2.6. Expression and Growth Association Analyses of a Potential Candidate Gene

A quantitative real-time polymerase chain reaction (qRT-PCR) analysis was conducted to explore the expression characteristics of a potential candidate gene during the following *A. japonicus* larval developmental stages: fertilized egg, blastula, gastrula, early auricularia larva, mid-auricularia larva, late-auricularia larva, doliolaria larva, pentactula larva, and juvenile [23,24]. Samples were treated with RNAwait (Solarbio, Beijing, China) for the subsequent RNA extraction [25]. Three tissues (body wall, respiratory tree, and intestine) were collected from three randomly selected individuals for the gene expression analysis. Total RNA was extracted from the collected tissues using a TaKaRa MiniBEST Universal RNA Extraction Kit (TaKaRa, Dalian, China) following the manufacturer’s instructions. The quality of the RNA samples was evaluated by 1.5% agarose gel electrophoresis and the concentrations were determined using the NanoDrop 2000 spectrophotometer. High-quality RNA (i.e., in terms of purity and integrity; OD_260_/OD_280_ = 1.8–2.0 and OD_260_/OD_230_ = 1.8–2.0) with a concentration greater than 50 ng/μL was used for the subsequent analysis. The reverse transcription reactions and qRT-PCR analyses were performed using the Prime Script™ RT reagent Kit (TaKaRa, Dalian, China) and the SYBR^®^ Premix ExTaq™ system TaKaRa, Dalian, China). The reference gene cytochrome b served as the internal control. Three replicates per sample were analyzed. Relative expression levels were calculated using the 2^−ΔΔCt^ method [26]. All the statistical analyses were performed using GraphPad Prism (version 9.0.0.121, GraphPad Software, San Diego, CA, USA). 

## 3. Results

### 3.1. Genotyping-by-Sequencing and SNP Discovery

The paired-end sequencing of the GBS libraries using the Illumina HiSeq 2500 platform generated 42.61 Gb raw data for 130 offspring (327,790 bp per individual) and the FP (853,903,540 bp) and MP (661,236,352 bp). After filtering the raw data, 42.02 Gb (offspring), 840,830,676 bp (FP), and 652,279,222 bp (MP) clean data were retained (i.e., data efficiency >98%). The quality of the sequencing data was high (Q20 and Q30 >90%) and the GC content was normal (>36%). Thus, the data were suitable for the subsequent analyses (Table S1). The use of *MseI*, *HaeIII*, and *MspI* resulted in 3,532,902 bp (FP), 2,740,669 bp (MP), and 176,567,947 bp (offspring) clean reads (Table S2).

Single nucleotide polymorphisms in the analyzed population were detected using the filtered BAM files. A total of 75,996 SNPs were detected in the FP, of which 2770 and 73,226 were homozygous and heterozygous SNPs, respectively. Additionally, 45,490 SNPs were detected in the MP, of which 16,403 and 29,087 were homozygous and heterozygous SNPs, respectively. A total of 48,675 polymorphic loci between the parents were detected. After filtering for genotyping completeness, the ‘lm × ll’ and ‘hk × hk’ categories comprised 2391 and 1521 SNPs, respectively (75% coverage), whereas the ‘nn × np’ category consisted of 1864 SNPs (66% coverage).

### 3.2. Genetic Linkage Map Construction

Both sex-specific linkage maps that were constructed included 22 LGs. The female map contained 3120 SNPs and spanned 2865.97 cM, with an average genetic distance of 0.92 cM. The genetic distance of each LG ranged from 20.58 cM (LG6) to 244.90 cM (LG1), and the number of markers in each LG ranged from 72 to 311, with the exception of LG6, which contained 20 SNPs (Table 1 and Table S3). The male map comprised 3814 SNPs and spanned 1746.60 cM, with an average genetic distance of 0.46 cM. The genetic distance of each LG ranged from 41.00 cM (LG21) to 116.99 cM (LG1), and the number of markers in each LG ranged from 115 to 265, with the exception of LG20 and LG21, which contained 44 and 36 SNPs, respectively (Table 1 and Table S3). The marker gap analysis revealed that 98.0% of the marker gaps in the male map were less than 5 cM and no marker gap was longer than 20 cM. In contrast, 95.8% of the marker gaps in the female map were less than 5 cM and one marker gap was longer than 20 cM (Table 2). These results indicated that the markers were distributed more evenly in the male map than in the female map.

**Figure 1:**
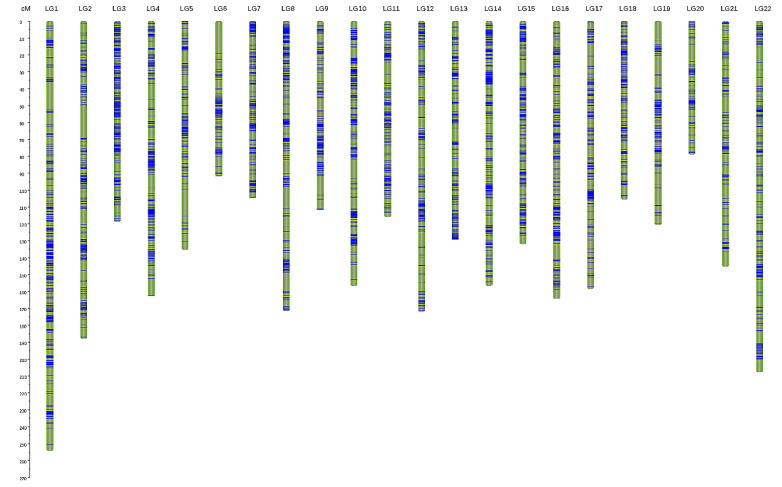
Consensus linkage map of *Apostichopus japonicus*. Blue bars represent SNPs. The scale on the left indicates the genetic distance in centimorgans (cM).

The sex-specific maps were integrated into a consensus map (Figure 1) with 22 LGs, which contained 6144 SNPs and spanned 3181.54 cM, with an average genetic distance of 0.52 cM. The genetic distance of each LG ranged from 78.95 cM (LG20) to 254.19 cM (LG1), and the number of markers in each LG ranged from 99 to 522. The marker gap analysis indicated that 98.5% of the marker gaps were less than 5 cM and no marker gap was longer than 20 cM. Thus, the markers were distributed more evenly in the consensus map than in the sex-specific maps (Table 1 and Table 2).

### 3.3. QTL Mapping and Growth Association Analysis

**Figure 2:**
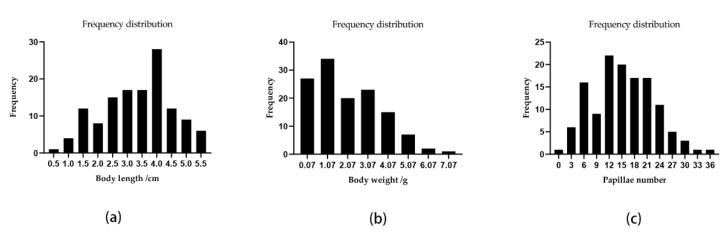
The frequency distribution of phenotypic data. (**a**) Body length; (**b**) body weight; (**c**) papillae number.

**Figure 3:**
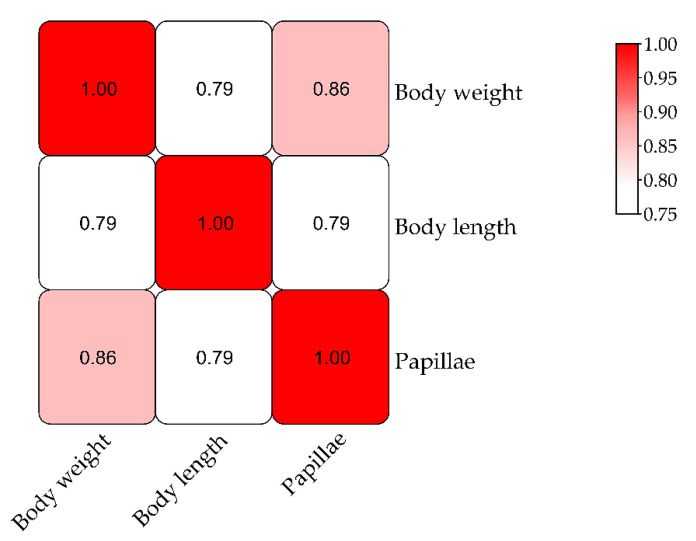
Correlations among three growth traits in *Apostichopus japonicus*.

A QTL mapping analysis was performed for *A. japonicus* growth-related traits (body weight, body length, and papillae number). The frequency distribution of phenotypic data is shown in Figure 2. The Pearson correlation analysis revealed that there were significant correlations among these three traits (Figure 3). The QTL mapping analysis involving the consensus map indicated that the QTLs for these three traits were all located close together in the same LGs (LG5, LG18, and LG22), which reflected the correlations among body weight, body length, and papillae number (Table 3 and Table S4). A total of 22 significant QTLs were detected with the LOD threshold set as 3.0. The most significant QTL related to body weight was located at 65.19 cM, presenting the highest LOD value of 5.06, explaining the highest percentage of the phenotypic variation (PVE) of 16.5 detected on LG18; that related to body length was located at LG5 (LOD 4.47, PVE 14.8%); and for papillae number was at LG18 (LOD 4.13, PVE 13.8%).

Eleven growth-related genes were identified from QTL mapping and the high-density genetic linkage map based on the *A. japonicus* genome annotation (Table 4 and Table S5). Among them, 8 genes were located in the genetic linkage region, including caveolin-1, homogentisate 1,2-dioxygenase (*HGD*), protein still life, isoforms C/SIF type 2 (*sif*), IgGFc-binding protein (*FCGBP*), nerve growth factor-like protein precursor (*NGF*), nucleoside diphosphate kinase 7, hyalin-like, neural cell adhesion molecule 1-like (*NCAM*), while 3 were in intergenic regions. The gene annotated as cyclic AMP-responsive element-binding protein 3-like protein 1 (*CREB3L1*), 150 bp upstream of the marker lm2694, was reported to be involved in growth hormone synthesis, secretion, and action. Transposon-derived Buster3 transposase-like protein (ZBED8) was 68 bp upstream of the marker lm575, and transcription factor 4 isoform X3 was located at 1239 bp upstream of marker np845. Among them, we noticed that the markers lm863 and lm864, which were both annotated to the *sif* gene on scaffold 157, had the highest PVE in body weight and papillae number, which means that they explain the highest percentage of the phenotypic variation, which suggested that *sif* may be a candidate growth-related gene. This gene encodes a guanine nucleotide exchange factor that is mainly related to the differentiation of nerve synapses.

### 3.4. Expression and Growth Association Analyses of a Potential Candidate Gene

**Figure 4:**
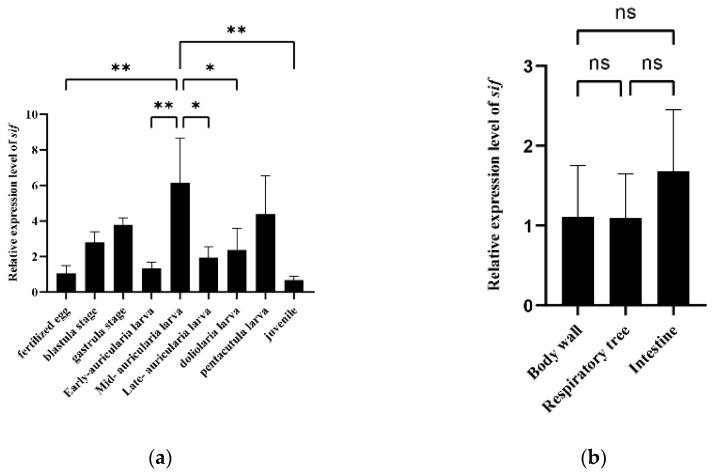
(**a**) Expression characteristics of the *sif* gene in different *Apostichopus japonicus* larval developmental stages. (**b**) Expression characteristics of the *sif* gene in different adult *Apostichopus japonicus* tissues. Asterisks indicate significant differences between the groups (* *p* < 0.05, ** *p* < 0.01).

To clarify the function of the *sif* gene, we performed qRT-PCR analysis to examine its level of expression at different larval developmental stages as well as in different tissues of adult specimens. The qRT-PCR primers are listed in Table S6. The qRT-PCR data indicated there were significant differences in expression among the examined larval developmental stages (*p* < 0.01). The relative expression of *sif* was highest in the mid-auricularia larval stage (6-times higher than the corresponding level in the fertilized egg stage) (Figure 4a). However, no significant differences were detected among the body wall, respiratory tree and intestine of adults (Figure 4b).

## 4. Discussion

Genetic linkage maps are a crucial resource for genetics and genomics research. In this study, we analyzed 130 F_1_ offspring and their parents using GBS technology to construct the first high-density, SNP-based genetic linkage map of sea cucumber (*A. japonicus*). The map was divided into 22 LGs, which is consistent with the results of an earlier investigation regarding *A. japonicus* chromosomes [1]. The established genetic linkage map provides a foundation for the mapping QTL associated with important quantitative and economically valuable traits as well as for inferring the genes responsible for these traits in *A. japonicus*.

A linkage map with a small marker interval (<2 cM), high sequence coverage, and uniform marker distribution may be called a high-density genetic map; such a map usually includes thousands of polymorphic markers [7,27]. The construction of a high-density genetic map requires a segregating population derived from genetically diverse parents with considerable number of polymorphisms. Commonly used populations in genomics research include F_1_, F_2_, backcross, doubled haploid, and recombinant inbred lines [28]. Because of the long generation time for sea cucumber, we selected the F_1_ population for this study. More specifically, a pair of unrelated sea cucumbers with large differences in weight were selected as the parents for the hybridization that produced the F_1_ population. In the past few years, microsatellites and AFLPs have been commonly used to construct genetic maps, most of which were low-density maps lacking sufficient and/or accurate information regarding the number and locations of genes or QTL. For example, a previously published *A. japonicus* genetic map constructed according to an F_1_ pseudo-testcross mapping strategy contained fewer than 200 sex-specific markers, with a marker density exceeding 7.0 cM [3]. Another *A. japonicus* genetic map, which was constructed on the basis of microsatellites and SNPs, consisted of approximately 200 markers, with a marker density of 7.0 cM [2]. Compared with traditional molecular markers, SNPs can be typed on a larger scale and they are generally more abundant in genomes, enabling analyses of all genomic regions [27]. To reveal sufficient sea cucumber genetic variation, we adopted GBS technology for genotyping. Compared with the RAD method, the GBS process is simpler because the single-well digestion of genomic DNA and adapter ligations help to minimize the required sample processing, resulting in relatively few DNA purification steps and no need for selecting fragment sizes. RAD-seq contains the step of ultrasonic random interruption, thus, the cost is higher than GBS, and RAD-seq is suitable for the study without reference genome [13]. By using the GBS approach, we constructed an *A. japonicus* genetic map 3181.54 cM in length, with 6144 SNPs and a map resolution of 0.52 cM. The number of markers and the resolution were substantially greater for our *A. japonicus* genetic map than for earlier maps constructed using more traditional markers.

In this study, the genetic distance was greater in the female map than in the male map, with a distance ratio of 1.64:1 (F:M), which was consistent with the results of previous investigations [2,4]. Regarding the species examined in earlier related studies, there were sex differences in the recombination rates of autosomes. As explained by Trivers [29], the recombination rate should be lower for males than for females because they have undergone stronger sexual selection; the meiosis in females occurs only during fertilization, and the chances of female haploid selection are relatively low. In the current study, an analysis of marker gaps detected 3738 gaps that were less than 5 cM in the male map (98.0% of all gaps), with no gaps longer than 20 cM. In contrast, there were 2978 gaps that were less than 5 cM in the female map (95.8% of all gaps), and one gap was longer than 20 cM. Additionally, the female map contained 99 gaps ranging from 5 to 10 cM and 21 gaps ranging from 10 to 20 cM, whereas the male map included only 43 gaps that were 5–10 cM and 12 gaps that were 10–20 cM. These findings imply that the male map was more uniform than the female map. A consensus map was constructed on the basis of the common markers between the MP and FP. The marker gap analysis of the consensus map indicated that 98.5% of the gaps were less than 5 cM, with no gaps greater than 20 cM. Hence, the consensus map was more uniform than the sex-specific maps.

The high-density genetic linkage map constructed enabled the mapping of QTL related to *A. japonicus* growth character. Growth traits are affected by multiple genes and are influenced by other factors. Moreover, they are among the most important traits affecting the economic value of *A. japonicus*. The genetic mechanism underlying growth has long been a topic of considerable interest among breeders. However, most research on sea cucumbers focused on stress-resistance traits, body color, and regeneration. In our study, we mapped the QTL associated with growth traits. The papillae number, as an important measure of the quality of sea cucumbers, affects the market price of sea cucumbers to a large extent [30], and is also an important breeding trait related to growth. Thus, we designated body weight, body length, and papillae number as three indicators to measure growth traits. Our analyses revealed the proximity of three growth-related QTL in LG5, LG18, and LG22. The results of Pearson’s association analysis and QTL mapping reflected the correlations among body length, body weight, and papillae number. Therefore, we may be able to improve the overall growth of *A. japonicus* by manipulating one of these traits. We annotated 11 candidate genes related to growth, which were mainly related to nerves, signal transduction, material transport, cell proliferation, and apoptosis. CREB3L1, is a membrane-bound transcription factor of the CREB/activating transcription factor (ATF) family, expressed in the glial cells, included in growth hormone synthesis/secretion [31], and is reported as may play an important role in limiting virus spread by inhibiting proliferation of virus-infected cells [32]. Caveolin-1 is an integral membrane protein soluble in multiple cellular compartments, and is reported to have functions in membrane trafficking, lipid transport, and signal transduction [33]. Nerve growth factor-like protein may act as a growth factor and participate in the paracrine regulation of prostate growth [34] and the regulation of spermatogenesis [35]. The neural cell adhesion molecule 1-like is one of the most classical cell adhesion molecules in vertebrates, and also is involved in regulating the trafficking of the neurotransmitter receptor, receptor-mediated signaling and behavior [36]. Protein still life, isoforms C/SIF type 2 gene, encodes the SIF protein, which is localized in the submembrane region. The SIF protein is believed to function as a guanine nucleotide exchange factor that controls synaptic differentiation through its effects on the organization of the actin cytoskeleton, possibly via the activation of Rho-like GTPases [37]. Moreover, it may mediate developmental processes. In our study, the *sif* gene was detected by the two markers (lm863 and lm864) with the highest PVE for body weight and papillae number, and it was located within the genetic linkage region. Therefore, we consider it to be an important growth-related candidate gene, and we carried out a preliminary exploration of the function of the *sif* gene. We found that *sif* was expressed during different *A. japonicus* larval developmental stages, especially in the mid-auricularia larval stage, when it reached its highest level of expression. Further investigations are needed to elucidate the precise regulatory effects of the SIF protein on *A. japonicus* embryonic development and growth.

## 5. Conclusions

In conclusion, we constructed the first SNP-based high-density genetic linkage map of sea cucumber (*A. japonicus*). The map (3181.54 cM) comprised 6144 SNPs distributed in 22 LGs, with an average genetic distance of 0.52 cM. The mapping of QTL for three growth-related traits (body weight, body length, and papillae number) suggested that the examined traits are correlated. Further, we identified 11 growth-related genes, among them the *sif* gene which we consider as an important candidate gene influencing sea cucumber growth. We preliminarily explored the spatiotemporal expression characteristics of this gene. Our research provides an important theoretical basis for the genetic analysis of growth traits and valuable genomic resources for the selection and genetic marker-assisted breeding of new varieties of sea cucumbers with excellent traits.

## Figures and Tables

**Table 1:** Summary of the characteristics of the consensus map and sex-specific genetic maps for *A. japonicus*.

LG	Consensus Map			Female Map			Male Map			F:M Distance Ratio (%)
	SNP Number	Distance (cM)	Average Distance (cM)	SNP Number	Distance (cM)	Average Distance (cM)	SNP Number	Distance (cM)	Average Distance (cM)	
1	522	254.19	0.487	311	244.897	0.787	268	116.991	0.437	2.09
2	282	187.96	0.667	138	175.923	1.275	188	93.965	0.500	1.87
3	400	118.72	0.297	190	112.690	0.593	230	75.942	0.330	1.48
4	339	162.74	0.480	179	154.192	0.861	202	90.616	0.449	1.70
5	194	135.31	0.697	93	124.056	1.334	115	71.007	0.617	1.75
6	134	91.77	0.685	20	20.580	1.029	119	91.482	0.769	0.22
7	261	104.68	0.401	72	57.992	0.805	216	79.304	0.367	0.73
8	397	171.5	0.432	207	178.561	0.863	240	67.782	0.282	2.63
9	251	111.47	0.444	122	95.650	0.784	171	71.104	0.416	1.35
10	374	156.63	0.419	145	131.186	0.905	265	106.481	0.402	1.23
11	218	116	0.532	123	92.466	0.752	125	76.28	0.610	1.21
12	274	172.04	0.628	144	164.174	1.140	174	96.972	0.557	1.69
13	247	129.08	0.523	135	119.459	0.885	151	64.097	0.424	1.86
14	377	156.6	0.415	183	158.806	0.868	230	64.863	0.282	2.45
15	324	132.01	0.407	160	125.678	0.785	208	76.551	0.368	1.64
16	339	164.2	0.484	183	157.621	0.861	210	90.283	0.430	1.75
17	247	158.4	0.641	144	153.737	1.068	151	82.783	0.548	1.86
18	218	105.59	0.484	109	98.316	0.902	143	77.2	0.540	1.27
19	183	120.61	0.659	82	70.275	0.857	124	86.103	0.694	0.82
20	99	78.95	0.797	82	81.608	0.995	44	43.736	0.994	1.87
21	136	145.33	1.069	136	151.049	1.111	36	40.997	1.139	3.68
22	328	207.76	0.633	162	197.049	1.216	204	82.058	0.402	2.40
Total	6144	3181.54	0.518	3120	2865.965	0.919	3814	1746.597	0.458	1.64

**Table 2:** Summary of the marker distance gaps (cM) per LG for *A. japonicus*.

LG ^1^	Consensus Map					Female Map					Male Map				
	<5 cM ^2^	5–10 cM	10–20 cM	>20 cM	Ratio (%) ^3^	<5 cM	5–10 cM	10–20 cM	>20 cM	Ratio (%)	<5 cM	5–10 cM	10–20 cM	>20 cM	Ratio (%)
1	516	5	1	0	98.9	297	11	3	0	95.5	264	4	0	0	98.5
2	275	5	1	0	97.9	130	5	2	0	94.9	184	3	0	0	98.4
3	398	1	0	0	99.7	186	3	0	0	98.4	226	3	0	0	98.7
4	333	4	1	0	98.5	172	4	2	0	96.6	199	2	0	0	99.0
5	189	3	1	0	97.9	85	6	1	0	92.4	112	2	0	0	98.3
6	130	2	1	0	97.7	17	2	0	0	89.5	114	2	2	0	96.6
7	259	1	0	0	99.6	71	0	0	0	100.0	213	2	0	0	99.1
8	390	4	2	0	98.5	197	5	4	0	95.6	238	0	1	0	99.6
9	246	3	1	0	98.4	115	5	1	0	95.0	167	3	0	0	98.2
10	369	3	1	0	98.9	137	6	1	0	95.1	261	0	3	0	98.9
11	215	2	0	0	99.1	118	4	0	0	96.7	122	2	0	0	98.4
12	266	7	0	0	97.4	133	10	0	0	93.0	169	3	1	0	97.7
13	244	2	0	0	99.2	131	2	1	0	97.8	149	1	0	0	99.3
14	374	2	0	0	99.5	178	4	0	0	97.8	228	0	1	0	99.6
15	320	3	0	0	99.1	153	4	2	0	96.2	206	1	0	0	99.5
16	335	3	0	0	99.1	177	4	1	0	97.3	206	3	0	0	98.6
17	241	5	0	0	98.0	137	5	0	1	95.8	147	3	0	0	98.0
18	215	2	0	0	99.1	106	2	0	0	98.2	141	1	0	0	99.3
19	176	3	3	0	96.7	78	2	1	0	96.3	117	4	2	0	95.1
20	97	0	1	0	99.0	77	3	1	0	95.1	41	1	1	0	95.4
21	129	6	0	0	95.6	129	6	0	0	95.6	32	3	0	0	91.4
22	322	5	0	0	98.5	154	6	1	0	95.7	202	0	1	0	99.5
Total	6039	71	13	0	98.5	2978	99	21	1	95.8	3738	43	12	0	98.0

^1^ LG, linkage group; ^2^ cM, centiMorgan; ^3^ Ratio, the number of markers < 5 cM divided by the total number of markers in the same LG.

**Table 3:** Summary of growth-related QTLs.

Trait	LG	Position	Marker	LOD ^1^	Variance	PVE (%) ^2^	Left Marker	Right Marker
Body weight	LG3	30.36	lm2808	3.13	2.54	10.6	lm2619	lm2621
	LG5	4.44	lm2694	3.19	2.54	10.8	lm3003	lm665
	LG6	52.98	lm829	3.79	2.48	12.6	lm1320	lm1656
	LG7	80.07	lm3265	3.1	2.54	10.5	lm2280	hk690
	LG7	90.37	np2126	3.33	2.52	11.2	np1854	np1510
	LG7	94.69	lm575	3.28	2.53	11	lm541	lm574
	LG18	47.37	hk521	3.15	2.54	10.6	lm2106	lm2035
	LG18	65.19	lm864	5.06	2.37	16.5	lm863	np586
	LG18	70.79	lm1135	3.9	2.47	13	np784	hk284
	LG22	198.7	np845	3.11	2.54	10.5	np1706	np837
Body length	LG5	1.6	lm2222	4.47	1.24	14.8	lm3000	lm3002
	LG5	4.44	lm2694	4.34	1.25	14.4	lm3003	lm665
	LG5	15.68	lm1384	3.73	1.28	12.6	hk711	lm1569
	LG18	65.08	lm863	3.5	1.29	11.8	hk520	lm864
	LG20	28.98	np2401	3.05	1.31	10.4	np787	np1754
	LG22	198.7	np845	3.06	1.31	10.4	np1706	np837
Papillae number	LG5	4.44	lm2694	3.57	43.36	12.1	lm3003	lm665
	LG6	53.03	lm832	3.95	42.78	13.2	lm1656	lm1834
	LG11	93.65	lm2134	3.85	42.93	12.9	lm1742	hk196
	LG18	53.03	lm2110	3.17	44	10.8	lm2111	lm2109
	LG18	65.08	lm863	4.13	42.5	13.8	hk520	lm864
	LG22	198.7	np845	3.52	43.45	11.9	np1706	np837

^1^ LOD, logarithm of odds; ^2^ PVE (%), the percentage of phenotypic variance explained.

**Table 4:** Summary of the growth-related candidate gene from QTLs.

Marker	LG	Scaffold	Annotation	GO			KEGG
				Cellular Component	Biological Process	Molecular Function	
lm2808	lg3	1093	-	-	-	-	-
lm2694	lg5	996	CREB3L1		transcription; transcription regulation; unfolded protein response;	activator; developmental protein; DNA-binding;	cAMP signaling pathway; growth hormone synthesis, secretion and action;
lm829	lg6	153	caveolin-1	endoplasmic reticulum membrane; Golgi membrane; caveolar macromolecular signaling complex; endocytic vesicle membrane;	apoptotic signaling pathway; cell differentiation; inactivation of MAPK activity; negative regulation of BMP and Wnt signaling pathway;	ATPase binding; signaling receptor binding; cell differentiation; regulation of canonical Wnt signaling pathway; molecular adaptor activity;	endocytosis; focal adhesion; transport and catabolism; genetic information processing;
lm3265	lg7	1691	-	-	-	-	-
np2126	lg7	1232	-	-	-	-	-
lm575	lg7	87	Transposon-derived Buster3 transposase-like protein	nucleoplasm;	protein binding;		signaling and cellular processes;
hk521	lg18	562	homogentisate 1,2-dioxygenase	cytosol; extracellular exosome; cytoplasm;	L-phenylalanine catabolic process; tyrosine catabolic process;	identical protein binding; metal ion binding;	alkaptonuria; tyrosine metabolism;
lm864	lg18	157	protein still life, isoforms C/SIF type 2	membrane; synapse;	actin cytoskeleton organization; signal transduction; cell junction; synapse formation	guanyl-nucleotide exchange factor activity;	signaling and cellular processes; Dbl-Like RhoGEF family proteins;
lm1135	lg18	233	-				-
np845	lg22	252	transcription factor 4 isoform X3				
lm2222	lg5	615	nerve growth factor-like protein	axon; dendrite; synaptic vesicle;	modulation of chemical synaptic transmission; nerve development; nerve growth factor signaling pathway;	growth factor activity; nerve growth factor receptor binding;	MAPK signaling pathway; calcium signaling pathway; cytokines and growth factors; intramolecular chaperones; apoptosis;
lm2694	lg5	996	CREB3L1				
lm1384	lg5	300	IgGFc-binding protein	extracellular matrix; extracellular exosome; extracellular space;			
lm863	lg18	157	protein still life, isoforms C/SIF type 2				
np2401	lg20	1900	nucleoside diphosphate kinase 7	mitochondrial inner membrane; mitochondrion;	lipid transport; nucleotide metabolism; transport; transcription regulation;	ATP binding; metal ion binding;	purine metabolism; membrane trafficking;
np845	lg22	252	transcription factor 4 isoform X3				
lm2694	lg5	996	CREB3L1				
lm832	lg6	153	hyalin-like	integral component ofmembrane;			
lm2134	lg11	576	neural cell adhesion molecule 1-like	cell membrane;	axonal fasciculation; cell adhesion; neurogenesis;	identical protein binding;	cell adhesion molecules;
lm2110	lg18	562	-	-	-	-	-
lm863	lg18	157	protein still life, isoforms C/SIF type 2				
np845	lg22	252	transcription factor 4 isoform X3				

## Data Availability

The raw sequencing data were archived at the NCBI Sequence Read Archive (SRA) under Accession Number PRJNA777474.

## Supplementary Materials

The following are available online at https://www.mdpi.com/article/10.3390/biology11010050/s1, Table S1: Information about samples for completed GBS sequencing; Table S2: Results about samples for enzyme digestion; Table S3: Information about the sex-specific genetic linkage map of *Apostichopus japonicus*; Table S4: Information about the QTL of growth traits; Table S5: Detailed information about the annotation of candidate genes related to growth; Table S6: Primers for qRT-PCR.
